# Giant ganglioneuroma of the mediastinum: a case report

**DOI:** 10.3389/fonc.2024.1408456

**Published:** 2024-10-16

**Authors:** Linfeng Song, Jiaren Zhang, Binlin Tian, Yongzhe Li, Xiaoyu Gu, Youlun Zhang, Lin Jiang

**Affiliations:** ^1^ Department of Radiology, The Third Affiliated Hospital of Zunyi Medical University (The First People’s Hospital of Zunyi), Zunyi, China; ^2^ Department of Pathology, The Third Affiliated Hospital of Zunyi Medical University (The First People’s Hospital of Zunyi), Zunyi, China

**Keywords:** ganglioneuroma, mediastinum, thoracic cavity, CT, MRI, case report

## Abstract

Ganglioneuroma (GN) is a rare benign neurogenic tumor that originates from the sympathetic nerves. It is extremely uncommon to find a lesion originating from the mediastinum that occupies the entire left hemithorax. In this report, we present the case of a 48-year-old female patient with a large mediastinal GN who presented with cough, sputum, and wheezing. Multislice spiral-enhanced CT and magnetic resonance imaging (MRI) revealed a large oval mass in the left thoracic cavity. The surgical operation completely resected the lesion, and the histopathological examination of the resected specimen confirmed the diagnosis of giant ganglion cell neuroma of the mediastinum. Due to the low incidence of GN and the lack of specific imaging manifestations, many radiologists may lack sufficient knowledge of GN and may be prone to misdiagnosis, resulting in delayed treatment. To enhance radiologists’ awareness of giant ganglion cell neuroma of mediastinal origin occupying the thoracic cavity, we provided detailed CT/MRI imaging information for this case, along with a brief summary of similar previously reported cases, to highlight the specific clinical and radiological features of this condition.

## Introduction

Ganglioneuroma (GN) is a rare benign neurogenic tumor that originates from primitive neural crest cells forming the sympathetic nervous system. It is primarily located in the paraspinal sympathetic ganglia and adrenal medullary region. Favorable sites for GN include the posterior mediastinum, retroperitoneal space, and adrenal gland (1–6), and there are few imaging descriptions of such cases. We present the case of a 48-year-old female patient with a giant mass in the left thoracic cavity, initially diagnosed as an isolated fibroma, but ultimately confirmed as a ganglioneuroma through histopathological analysis. Therefore, we provide the CT and MRI images to raise awareness among clinicians and radiologists of this rare type of tumor, helping to avoid misdiagnosis and delayed treatment.

## Case presentation

**Figure 1:**
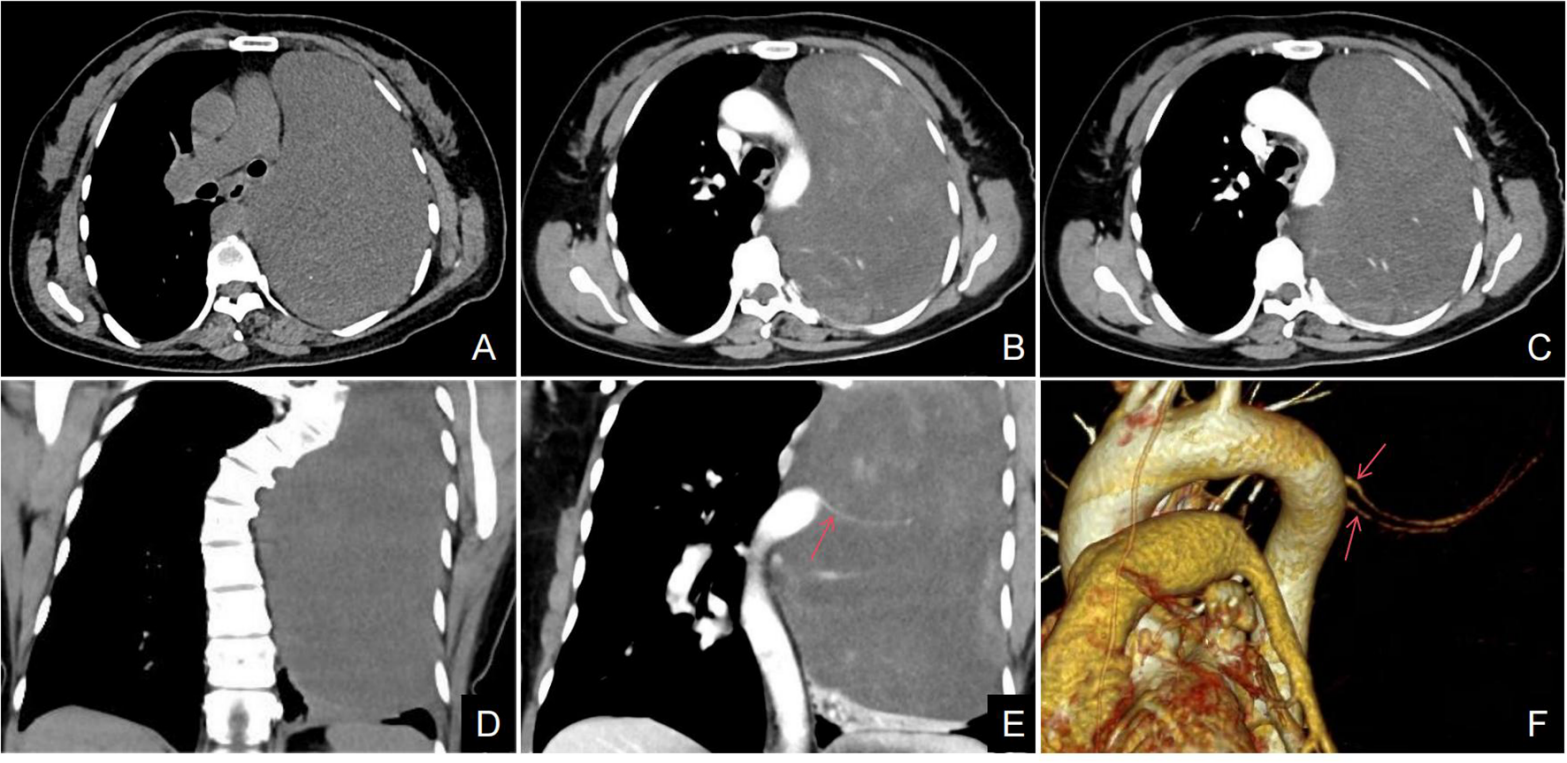
**(A–F)** Axial CT scan, a large well-defined mass with punctate calcifications was seen in the thoracic cavity **(A)**. The axial CT enhancement image shows uneven delayed enhancement of the mass with a “patchy” appearance **(B, C)**; the arterial phase of the coronary CT and VR (red arrow) clearly shows that the trophoblastic vessels of the mass are from the abdominal aorta **(E, F)**. Coronal CT scan showed scoliosis in relation to the tumor **(D)**.

**Figure 2:**
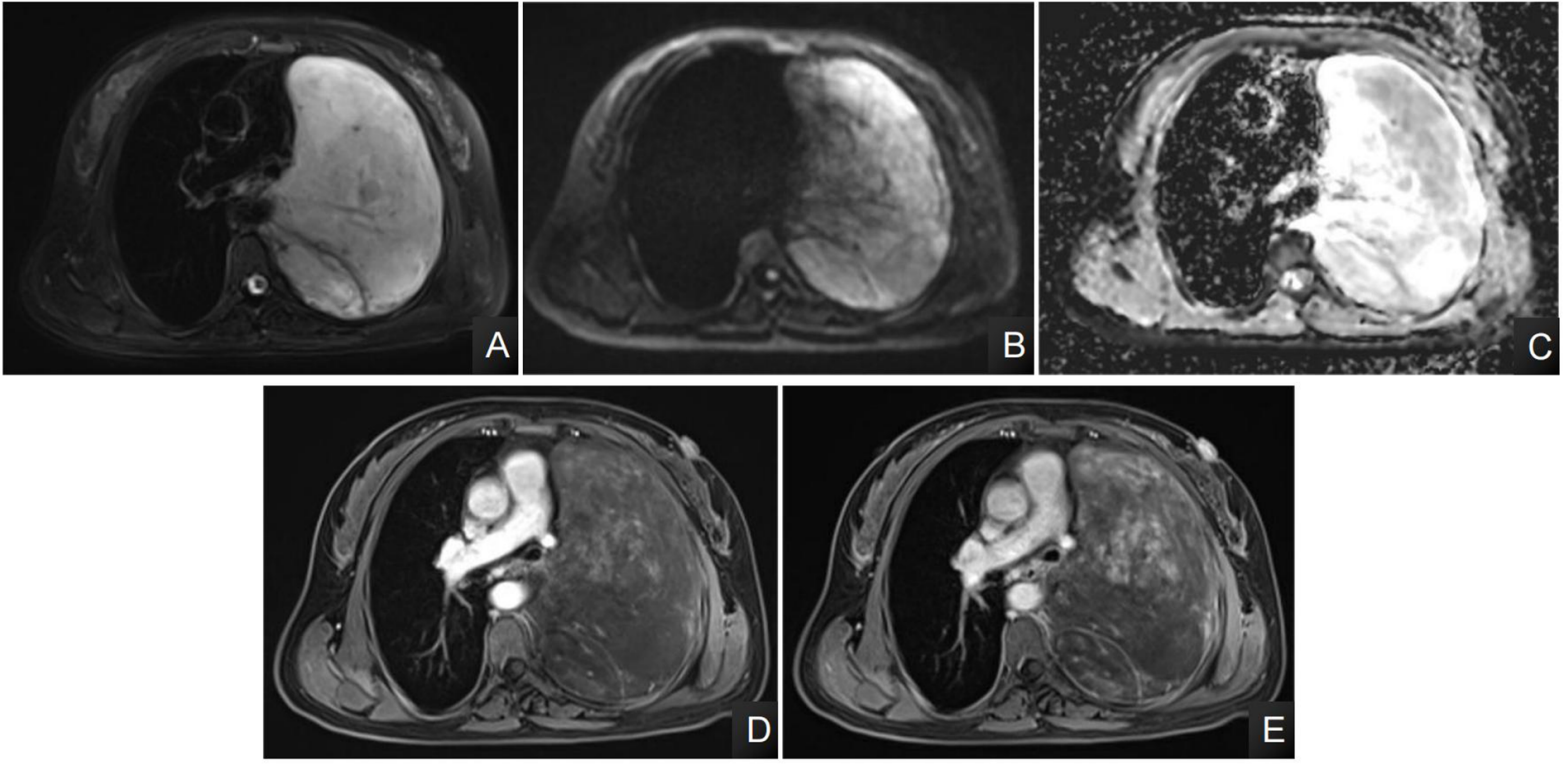
**(A–E)** The axial T2-weighted MR images reveal an uneven high signal with a “streaky” appearance **(A)**; DWI images showed partially high signal, and ADC images showed a slightly low signal in the ADC in the area corresponding to the high signal in DWI **(B, C)**. The axial MRI enhancement image showed mild to moderate heterogeneous delayed enhancement of the lesion **(D, E)**.

**Figure 3:**
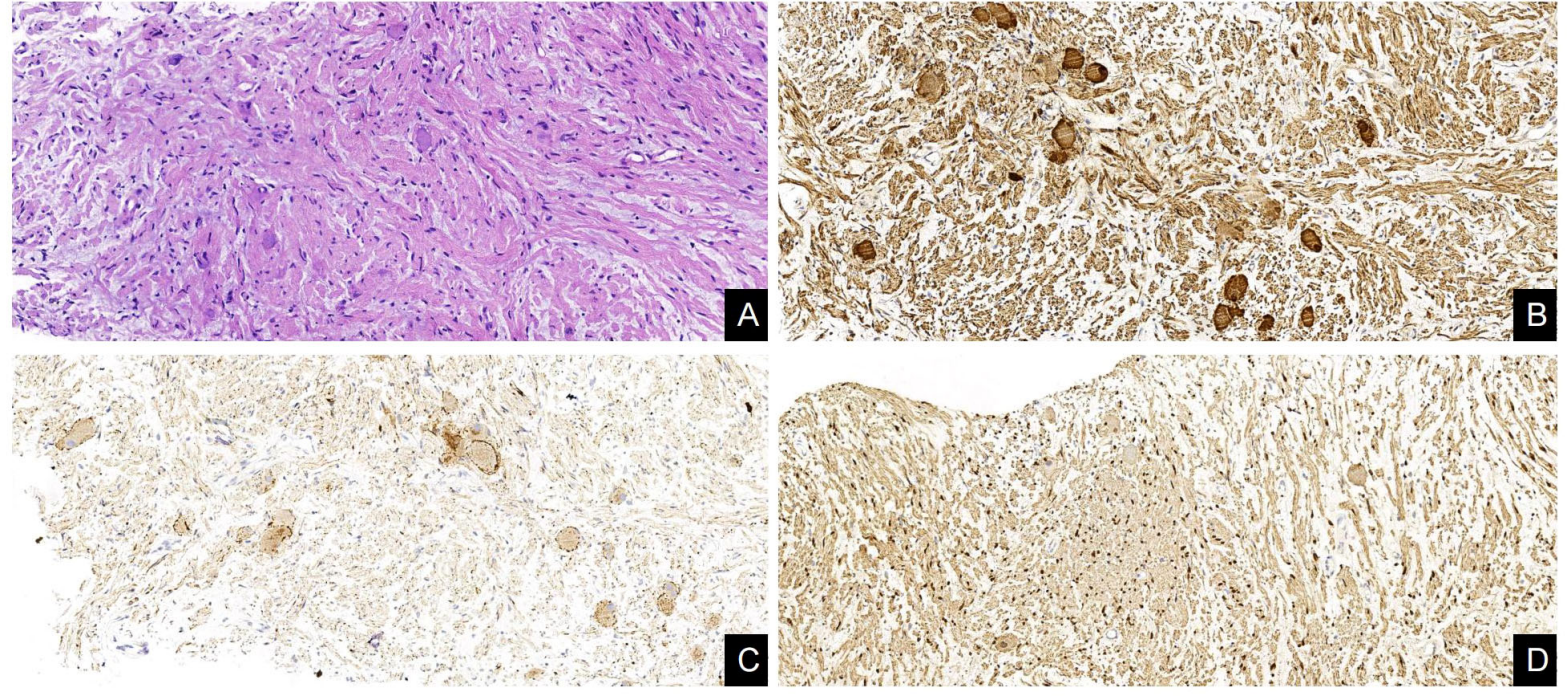
Histological and immunohistochemical features of GN. **(A)** The distribution of mature ganglion cells and spindle cells (proliferating nerve sheath cells and nerve fibers) was diffuse, as shown by the Hematoxylin-eosin staining. **(B–D)** Immunohistochemical staining showed that the ganglion cells expressed NSE(+), Syn(+), and Schwann cells expressed S-100(+). [Original magnifications: **(A–D)** 200×].

A 48-year-old female patient was admitted to our hospital due to coughing and sputum for over a month without any apparent triggers. The patient also experienced hot flashes, night sweats, poor appetite, and a weight loss of 2.5 kg in the past week. Treatment at an outpatient clinic was ineffective, leading to the admission to our hospital. The left lung’s respiratory examination revealed solid sounds on percussion, hypopnea, and decreased lung function. The rest of the examination was unremarkable, and laboratory results were normal. The CT scan revealed a well-defined elliptical mass in the left mediastinum and thoracic cavity, measuring 18.3×15.2×11.0 cm. The mass had clear borders and contained coarse granular and punctate calcifications. The CT value of the mass was about 25 HU, which was lower than the density of the muscle tissue (**Figure 1A**), enhanced with heterogeneous enhancement (**Figures 1B, C**). Additionally, the lesion has secondary effects leading to a scoliosis deformity of the thoracic spine (**Figure 1D**), and the artery supplying blood to the tumor was emanating from the thoracic aorta (**Figures 1E, F**). The MRI revealed a shadow in the left thoracic cavity on T1WI, the lesion mostly appears as low or slightly low signal intensity, while on T2WI, it shows heterogeneous high signal intensity with linear or streaky low signal intensities within the high signal area (whorled or stripe sign) (**Figure 2A**). The DWI portion of the tumor showed a slightly high signal (**Figure 2B**), and the corresponding ADC signal was slightly attenuated (**Figure 2C**). The enhancement scan showed inhomogeneous delayed enhancement (**Figures 2D, E**). The preoperative diagnosis includes Solitary Fibrous Tumors (SFT) and Sarcoma. During the surgery, the patient had a large tumor in the left thoracic cavity removed. The tumor occupied about 90% of the cavity, but did not adhere to surrounding tissues or organs. The surgeon was able to remove the tumor completely, including the paraspinal root lesion, and observed three trophoblastic vessels emanating from the aorta, confirming that the tumor originated from the mediastinum. The histopathological findings revealed a low-grade soft tissue tumor. The distribution of mature ganglion cells and spindle cells (proliferating nerve sheath cells and nerve fibers) was diffuse, as shown by the Hematoxylin-eosin staining (**Figure 3A**). Immunohistochemical staining showed positive results for ganglion cell calretinin, Vimentin, GFAP, NSE (**Figure 3B**), PGP9.5, S-100 (**Figure 3D**), and Syn (**Figure 3C**), while Lambda was negative. Additionally, the Ki-67 proliferation index was low (1%).

## Discussion

GN accounts for approximately 0.1%-0.5% (7) of neurological tumors, the most common (8) sites of GN are the retroperitoneum and posterior mediastinum, and can occur at any age, with a prevalence in young and middle-aged adults, with no difference between the sexes (9). GN is slow-growing and asymptomatic. Most tumors are large in size at the time of discovery. A retrospective study by Zhang (10) et al. on the imaging of 35 patients with retroperitoneal GN found that the average tumor size was 10.12 ± 4.56 cm. According to the current literature review, this case is one of the largest mediastinal GNs reported in adults to date (11, 12). Large tumors can compress nearby organs, leading to discomfort, pain, coughing, chest tightness, and other clinical symptoms (13). Tumors originating from primitive neural crest cells of the sympathetic nervous system can be classified into three categories based on their degree of differentiation. GN, ganglioneuroblastoma (GNB), and neuroblastoma (NB) are three types of tumors (14). GN is a well-differentiated tumor that consists of ganglion cells, mature nerve fibers, Schwann cells, and mucus stroma. It exhibits benign biological behaviors, and the diagnosis depends on the observation of ganglion cells. Microscopically, GN often shows a scattered distribution of mature ganglion cells within the nerve fiber tissue. It is positive for immunohistochemical staining for S100, Vim, NSE, and myelin basic protein (14, 15).

Mediastinal GN typically presents as a well-defined soft tissue mass, often involving the capsule or pseudocapsule. In this case, the peritoneum was clearly demonstrated on MRI. The density or signal of GN is related to the proportion of mucus matrix, nerve fibers, and ganglion cells (16). When the mucus matrix component in the GN is high, the CT scan density is lower than that of the muscle, and the T2WI shows a high signal. When the ganglion cells and nerve fibers increase, the CT density increases, and the T2WI shows a medium or slightly high signal (17). GN is characterized by a ‘swirling’ appearance on MR scans, which is caused by the interspersed Schwann cells and nerve fibers within the tumor. However, it is important to note that the probability of observing this sign varies among studies. Zhang et al. (10) reported that up to 73.7% of GNs had a ‘swirling’ appearance on T2WI and/or CT. In the present case, the tumor exhibited a striated low signal on T2WI, which is consistent with the findings of Luo et al. (18) named this feature the ‘streak sign’ and suggested that it shares a similar pathological mechanism with the ‘swirl sign’. These features can assist radiologists in improving the accuracy of diagnosing and identifying GN, especially in cases with atypical imaging presentations. Additionally, they can better aid clinicians in assessing the nature of the lesions and their potential prognosis, thereby facilitating the development of more effective treatment plans. DWI indirectly reflects the degree of diffusive movement of intracellular and extracellular water molecules. Most GNs exhibit high signal on DWI (18–20), which is related to their intra-tumor enrichment of mucus matrix. Calcifications were found in approximately 41-64.6% of GNs (21, 22), with fine punctate calcifications being the most common, followed by linear calcifications, consistent with literature reports (9). MRI-enhanced scans were performed in this case. The degree and/or extent of enhancement gradually increased over time, which is consistent with Kato et al’s study (23). MRI-enhanced scans provide higher soft-tissue contrast, making it easier to determine the degree of enhancement of the lesion. They are more valuable than CT-enhanced scans for diagnosing GN. Additionally, the literature reports a statistically significant correlation between tumor size and degree of enhancement (18); Specifically, about 55.17% of tumors with a diameter of < 5cm exhibit mild enhancement, while 77.27% of tumors with a diameter of≥5cm or greater show no significant enhancement; the degree of enhancement is determined by the proportion of mucus stroma (no enhancement), ganglion cells, and mesenchymal blood vessels (little enhancement) in the tumor. Most tumors show mild delayed enhancement of intra-lesional strips and intervals, which is caused by the large amount of mucus stroma in the tumor. This leads to the enlargement of extracellular spaces that can cause contrast retention (24).

The patient’s condition was diagnosed as SFT and sarcoma. The lesion was large, approximately 18.3 cm in diameter, compressing surrounding lung tissue, bronchi, and large mediastinal vessels, On T2-weighted MRI, the lesion appeared to contain mucinous and fibrous components, and subsequent CT/MRI enhancement scans of the tumor showed delayed enhancement and the blood supply artery was found to originate from the mediastinal aorta, consistent with an extrapulmonary lesion; the imaging performance of SFT and sarcoma is similar to that of intrathoracic space-occupying lesions, making it difficult to differentiate between the three. However, on CT scanning, the density of the tumor is more homogeneous throughout the day, and SFT and sarcoma are prone to necrosis and cystic degeneration (25); The SFTs originate from mesenchymal cells located in the subepithelial tissue of the pleura (26), are often adjacent to the pleura, and enhanced SFTs may show tortuous vascular shadows (27). Sarcoma is a malignant tumor originating form multipotential mesenchymal tissue (28); It has high invasiveness, often invading adjacent lung tissue and peripheral blood vessels (29), lesions prone to hemorrhage and necrosis commonly show a “triple sign” on T2-weighted imaging (30); in this case, T2-weighted imaging was not present, and there were changes to the surrounding structures due to nudging and compression. Secondly, it is important to differentiate intrathoracic GN from schwannoma, neurofibromas, GNB, NB, and other neurogenic tumors. Schwannoma have a tendency to cystic degeneration and hemorrhage, enhanced imaging shows partial significant enhancement, and the lesions often invade the spinal canal through the intervertebral foramina, resulting in a “dumbbell-shaped” change with enlarged foramina, and the transverse diameter of the lesion is greater than the longitudinal diameter, Lin (31) et al. found that the ratio of transverse to longitudinal diameter was smaller in GN than in nerve sheath tumors. Neurofibromas are frequently present in neurofibromatosis. They typically exhibit uniform soft tissue density and mild to moderate enhancement on enhancement scans (32). According to Wang (33) and other researchers, radiomics features showing a target sign on T2WI, in conjunction with the patient’s age at the time of initial diagnosis, can differentiate between NB and GNB/GN. This differentiation can aid in the identification of the pathology of peripheral neuroblastoma in children. Neuroblastoma, GNB, and NB are more common in pediatrics and adolescents, with a younger age of onset. They are prone to hemorrhage, necrosis, and calcification, and marked by inhomogeneous enhancement. Neuroblastoma is highly malignant and prone to invade surrounding structures and distant metastasis.

## Conclusion

In summary, we present a case report of a rare occurrence of neurogenic tumor in the mediastinum and thoracic cavity. Thoracic neurogenic tumors are benign and uncommon, with a higher prevalence in children and young adults. The imaging characteristics of these tumors include homogeneous hypodensities with punctate calcification in CT scans, inhomogeneous high signals with hairy cords in T2WI, and low signals in MRI scans. The arterial phase shows no or mild enhancement, while the delayed phase exhibits progressive and inhomogeneous enhancement. In cases where the tumor is larger, it can displace or encircle surrounding blood vessels, ribs, and other structures. Imaging plays a crucial role in the diagnosis, differential diagnosis, and treatment of this disease.

## Data Availability

The original contributions presented in the study are included in the article/supplementary material. Further inquiries can be directed to the corresponding author.
